# Serological response to vaccination in post-acute sequelae of COVID

**DOI:** 10.1186/s12879-023-08060-y

**Published:** 2023-02-16

**Authors:** Sandy Joung, Brittany Weber, Min Wu, Yunxian Liu, Amber B. Tang, Matthew Driver, Sarah Sternbach, Timothy Wynter, Amy Hoang, Denisse Barajas, Yu Hung Kao, Briana Khuu, Michelle Bravo, Hibah Masoom, Teresa Tran, Nancy Sun, Patrick G. Botting, Brian L. Claggett, John C. Prostko, Edwin C. Frias, James L. Stewart, Jackie Robertson, Alan C. Kwan, Mariam Torossian, Isabel Pedraza, Carina Sterling, Caroline Goldzweig, Jillian Oft, Rachel Zabner, Justyna Fert-Bober, Joseph E. Ebinger, Kimia Sobhani, Susan Cheng, Catherine N. Le

**Affiliations:** 1 Department of Cardiology, Smidt Heart Institute, Cedars-Sinai Medical Center, Los Angeles, CA USA; 2 Cardiovascular Division, Brigham and Women’s Hospital, Boston, MA USA; 3 David Geffen School of Medicine, University of California Los Angeles, Los Angeles, CA USA; 4 Abbott Diagnostics, Abbott Park, IL USA; 5 Division of Infectious Diseases, Department of Medicine, Cedars-Sinai Medical Center, Los Angeles, CA USA; 6 Division of Pulmonary and Critical Care Medicine, Cedars-Sinai Medical Center, Los Angeles, CA USA; 7 Cedars-Sinai Medical Care Foundation, Cedars-Sinai Medical Center, Los Angeles, CA USA; 8 Department of Pathology and Laboratory Medicine, Cedars- Sinai Medical Center, Los Angeles, CA USA

**Keywords:** SARS-CoV-2, Post-acute sequelae, Immune activation, Serological response, Anti-spike antibody

## Abstract

**Background:**

Individuals with post-acute sequelae of COVID (PASC) may have a persistence in immune activation that differentiates them from individuals who have recovered from COVID without clinical sequelae. To investigate how humoral immune activation may vary in this regard, we compared patterns of vaccine-provoked serological response in patients with PASC compared to individuals recovered from prior COVID without PASC.

**Methods:**

We prospectively studied 245 adults clinically diagnosed with PASC and 86 adults successfully recovered from prior COVID. All participants had measures of humoral immunity to SARS-CoV-2 assayed before or after receiving their first-ever administration of COVID vaccination (either single-dose or two-dose regimen), including anti-spike (IgG-S and IgM-S) and anti-nucleocapsid (IgG-N) antibodies as well as IgG-S angiotensin-converting enzyme 2 (ACE2) binding levels. We used unadjusted and multivariable-adjusted regression analyses to examine the association of PASC compared to COVID-recovered status with post-vaccination measures of humoral immunity.

**Results:**

Individuals with PASC mounted consistently higher post-vaccination IgG-S antibody levels when compared to COVID-recovered (median log IgG-S 3.98 versus 3.74, P < 0.001), with similar results seen for ACE2 binding levels (median 99.1 versus 98.2, P = 0.044). The post-vaccination IgM-S response in PASC was attenuated but persistently unchanged over time (P = 0.33), compared to in COVID recovery wherein the IgM-S response expectedly decreased over time (P = 0.002). Findings remained consistent when accounting for demographic and clinical variables including indices of index infection severity and comorbidity burden.

**Conclusion:**

We found evidence of aberrant immune response distinguishing PASC from recovered COVID. This aberrancy is marked by excess IgG-S activation and ACE2 binding along with findings consistent with a delayed or dysfunctional immunoglobulin class switching, all of which is unmasked by vaccine provocation. These results suggest that measures of aberrant immune response may offer promise as tools for diagnosing and distinguishing PASC from non-PASC phenotypes, in addition to serving as potential targets for intervention.

**Supplementary Information:**

The online version contains supplementary material available at 10.1186/s12879-023-08060-y.

## Background

Many individuals infected by COVID continue to experience symptoms persisting more than 12 weeks beyond the acute illness [1]. The pathophysiology underlying this morbid syndrome, also referred to as long-haul COVID or post-acute sequelae of COVID (PASC), [2] remains unclear. Aberrant cellular and humoral responses following original infection have been hypothesized as the predominant drivers of persistent symptoms in PASC. Proposed mechanisms include cross-reactivity and molecular mimicry triggering autoimmunity, delayed viral clearance leading to chronic inflammation and immune exhaustion, alternations in microbiota, and impaired immune-metabolism [3]. A common element of most proposed etiologies is the potential role of excess humoral activation. One accessible method for assessing humoral activation is to evaluate serological profiles following the planned administration of COVID vaccination. If found among patients with PASC, a distinctive pattern of antibody response to vaccine provocation could serve as a readily available diagnostic and prognostic tool for clinicians – as well as a step towards clarifying putative underlying immune mechanisms. To this end, we examined the extent to which administration of vaccinations could elicit a serological response profile that differentiates individuals with PASC from those with complete recovery from COVID without residual sequelae.

## Methods

### Source cohorts

All participants included in the current analyses were adults who were enrolled into observational cohort studies beginning in September 2020, each with a parallel longitudinal study design centered on repeated assessments of SARS-CoV-2 serology, exposures, and outcomes (Figure S1). Individuals with PASC were identified from a patient-based cohort of individuals diagnosed with PASC and treated clinically for the condition by specialist providers, as detailed below. Individuals with recovered COVID were identified from a healthcare worker cohort of individuals with engaged in ongoing study protocols while employed in our healthcare system; individuals without prior COVID infection were identified as an additional referent sample from this same source cohort. All research was performed in accordance to the Declaration of Helsinki and Cedars-Sinai institutional review board; all study participants provided written informed consent for all protocols, which were reviewed and approved by the Cedars-Sinai institutional review board.

***PASC cohort.*** This cohort included adult patients enrolled into a longitudinal study of COVID risks and outcomes while receiving medical care for PASC in our COVID Recovery Program clinic at Cedars-Sinai Medical Center in Los Angeles, California. All study cohort participants had medically confirmed prior COVID diagnosis and had physician assessed ongoing symptoms persisting beyond 12 weeks from the index diagnosis. In addition to the clinical evaluations, participants completed standardized surveys on COVID related exposures as well as post-infection symptoms and functional status at the initial visit and at serial timepoints over the course of the study. At the time of study enrollment and at follow-up study visits, plasma samples were collected for the serological assays described below. For the current analysis, we identified 463 adult patients enrolled as of February 11, 2022, of which a total of 247 participants had complete clinical data and had provided at least 1 blood sample for serological assays within 24 weeks of receiving any initial vaccination dose. Of this sample, we excluded 2 patients due to having received pre-exposure monoclonal antibody treatment (given its potential to markedly increase antibody levels in the absence of infection or additional vaccine dosing). Thus, the final sample of PASC patients for the current analysis was 245 individuals with non-missing data on key covariates including infection timing, clinical, and serological measures (Figure S1).

***COVID recovered cohort.*** The referent cohort was derived from a longitudinal cohort study of healthcare workers who received vaccination with BNT162b2 at Cedars-Sinai Medical Center, with study design and sampling procedures detailed previously [4, 5]. Participants completed surveys on exposures and symptoms at serial timepoints over the course of the study. To verify self-reported absence or presence of comorbidities for study participants, medical charts were reviewed via the electronic health record [5]. For the current analysis, we identified from 1751 adult participants enrolled as of February 11, 2022, of which a total of 1029 participants had complete clinical data and had provided at least 1 blood sample for serological assays within 24 weeks of receiving any initial vaccination dose. Of this sample, we excluded 62 participants due to having documented or reported interval COVID infection within 24 weeks of receiving initial vaccination. Of this sample, 238 participants reported having COVID preceding the initial vaccination without any clinical sequelae; we excluded 152 participants from this subset given missing data on the timing of the prior COVID infection, leaving a final sample of 86 participants with confirmed recovered COVID and non-missing data on key covariates including infection timing, clinical, and serological measures (Figure S1).

***Referent cohort.*** Of the 1029 participants identified from the healthcare worker cohort sampling above, after excluding 238 participants who had reported COVID prior to initial vaccination and the 62 participants who developed COVID within 24 weeks after initial vaccination, there remained 729 participants with no documented or reported COVID through the 24 weeks following initial vaccination. These 729 participants were available for the current analysis as the referent cohort with no prior COVID and non-missing data on key covariates including clinical and serological measures (Figure S1).

### Serology

Serological assays for antibodies to the receptor binding domain of the S1 subunit of the viral spike protein (IgG-S and IgM-S) and nucleocapsid (IgG-N) were performed using the Abbott SARS-CoV-2 IgG II assay (Abbott Labs, Abbott Park, IL) [6]. Serological measurements were taken from distinct plasma samples (i.e. no measurements were repeatedly measured from the same sample). For PASC patient participants of the study, antibody levels were measured from plasma samples collected at initial and follow-up clinic visits. For the healthcare worker study participants, antibody levels were measured from plasma samples collected at pre-specified time points before and after vaccination as previously described [4, 7]. A high-throughput angiotensin-converting enzyme 2 (ACE2) binding inhibition assay was also used to directly assess viral neutralization. The assay measures the presence of IgG-S antibodies that bind to ACE2 receptors and has been shown to be highly correlated to plaque reduction neutralization tests as well as IgG-S assays [4]. While prioritizing the IgG-S assay, the additional serological assays were performed on as many samples collected as permissible based on the availability of sample volume and resources (e.g. reagents) (Table S1).

### Clinical assessments

We determined history and dates of prior SARS-CoV-2 infection based on concordance of clinical data documented in health records, elevated IgG-N (index ≥ 1.4), [8] and the self-reported survey information collected [9, 10]. All cases of data discrepancy regarding prior SARS-CoV-2 infection status underwent algorithmic and manual physician adjudication, including medical chart review for evidence of positive SARS-CoV-2 PCR or antibody testing that could have been resulted by outside institutions. All participants included in the current analyses were adults who were enrolled into an observational cohort study beginning in September 2020; for participants in both the PASC and COVID-recovered groups, the timing of prior index infections was distributed across periods dominated by different SARS-CoV-2 variants (Figure S2).

### Statistical analyses

Antibody measurement values were log10 transformed if confirmed to demonstrate non-normal distribution (i.e. IgG-S and IgM-S). We used Wilcoxon Rank Sum tests to primarily compare antibody levels between the two prior COVID infected cohorts: individuals with PASC and individuals recovered from prior COVID without PASC. We secondarily compared antibody levels in PASC with levels in a referent cohort of individuals with no prior COVID. We first transformed the time from vaccination variable using natural cubic splines, with knot placement optimized at the 5th, 35th, 65th, and 95th percentiles. We used spline transformation for the time from vaccination variable given the observation that longitudinal patterns of change over time for post-vaccination antibody levels are not sufficiently represented by alternate approaches to transformation that could be considered for statistical modeling [7]. For the primary pre-specified analyses, we then used multivariable linear regression analyses (adjusting for time from vaccination, age, sex, race/ethnicity, and indices of comorbidity burden) to examine the associations of PASC versus COVID-recovered status with first post-vaccination level of log_10_ IgG-S for each participant assayed more than 8 weeks after vaccination during the ‘plateau’ period [7]. We conducted sensitivity analyses excluding individuals who received the Johnson & Johnson (J&J) vaccine, given prior reports of lower antibody response following J&J compared to mRNA vaccination [11]. We repeated the main analyses for available data for IgM-S, IgG-N, and ACE2 measurements. In secondary analyses, we repeated regression analyses including multiplicative interaction terms for age and sex to assess for potential effect modification of PASC versus COVID-recovered group on the primary outcome (i.e. IgG-S antibody level during the ‘plateau’ period). Using all measurement data available across all timepoints for the IgG-S, IgM-S, and ACE2 assays (Table S1), we also secondarily used ROC analyses to examine the extent to which each assay may perform as a tool for distinguishing PASC from recovered-COVID status. We conducted all statistical analyses using R (v4.0.4) and considered statistical significance as a two-tailed P value < 0.05.

## Results

The primary cohort of 245 individuals with PASC were initially evaluated at a median 260 (IQR 199, 361) days following infection. The demographic and clinical characteristics for participants included in the primary analyses including both individuals in the PASC cohort and individuals in the COVID-recovered cohort are shown in Table 1, with PASC symptoms-related characteristics shown in Table S2; characteristics of the referent sample with no prior COVID are shown in Table S3. Participants had IgG-S, IgM-S, and IgG-N antibodies and ACE2 binding capacity assayed before or after receiving COVID vaccination of types shown in Table 1, with the range of days between completed vaccination and timing of serial antibody assays found to be similar across participant groups: -155 to + 363 days for PASC, -415 to + 384 days for COVID-recovered, and − 368 to + 483 for no prior COVID.

**Table 1:** Characteristics of the primary study samples

Characteristic	Prior COVID		P value
	PASC	Recovered COVID	
N	245	86	
Age in years, mean (SD)	48.7 (13.5)	42.0 (11.8)	< 0.001
Age in years, range	22 to 79	23 to 76	
Male, n (%)	81 (33)	23 (27)	0.34
Non-Hispanic White, n (%)	124 (51)	34 (40)	0.10
Comorbidities,* n (%)			
Autoimmune disorder	19 (8)	2 (2)	0.13
Cancer	15 (6)	3 (3)	0.52
Chronic Pulmonary Disease	1 (0)	8 (9)	< 0.001
Diabetes Mellitus	23 (9)	3 (3)	0.13
Hypertension	53 (22)	10 (12)	0.06
Elixhauser score, mean (SD)	0.8 (2.9)	0.7 (2.4)	0.77
Hospitalized for COVID-19, n (%)	54 (22)	0 (0)	< 0.001
Post-exposure monoclonal antibody infusion, n (%)	15 (6)	1 (1)	0.87
Days between infection onset and vaccination,† median [IQR]	131 [106, 242]	194 [63, 273]	0.12
SARS-CoV-2 vaccine type received, n (%)			< 0.001
Pfizer (monovalent)	125 (51)	86 (100)	
Moderna (monovalent)	64 (26)	0 (0)	
Johnson & Johnson	22 (9)	0 (0)	
Other/Unknown	34 (14)	0 (0)	

*Comorbidities were derived from the electronic medical record using previously validated Elixhauser definitions

†Vaccination is defined as the date of vaccine completion: second dose of a two-dose regimen, or dose of a single-dose regimen

**Fig. 1:**
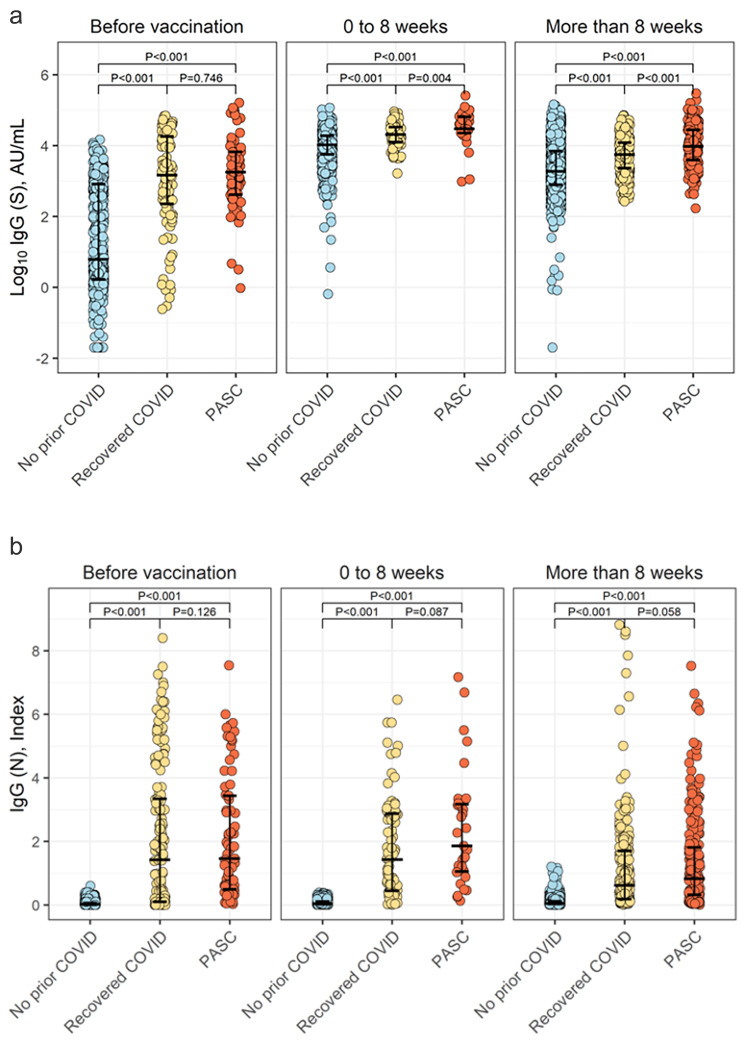
Pre- and post-vaccination IgG-S and IgG-N antibody levels in PASC. Individuals with PASC had a higher IgG-S antibody response to COVID vaccination compared to that seen in COVID-recovered or never-infected individuals (Panel A), suggesting more pronounced immune activation. Differences in vaccination provoked IgG-S response persisted over time despite PASC-affected and COVID-recovered individuals having similar levels of IgG-N antibody levels (Panel B), a marker of severity and timing of prior exposure to natural infection

In primary analyses comparing between the prior COVID infected groups, individuals with PASC compared to recovered COVID had similar pre-vaccination IgG-S antibody levels (Table 2) but mounted a higher post-vaccination IgG-S antibody response (median log IgG-S 3.98 versus 3.74, P < 0.001) assessed more than 8 weeks after vaccination (Fig. 1); parallel results were observed as early as during the initial 0–8 week period following vaccination. There was no significant inter-group difference in the time interval between infection and vaccination (P = 0.12, Table 1) or IgG-N levels (Table 2; Fig. 1), considered an indicator of timing and severity of prior COVID illness [12]. Similar trends were observed in multivariable analysis adjusted for age, sex, race/ethnicity, and indices of comorbidity burden (Table 3); results were also similar with even larger estimated magnitudes of difference in sensitivity analyses that excluded individuals who received the J&J vaccine (i.e. excluding 22 individual J&J vaccine recipients in the PASC cohort).

**Table 2:** Serological measures before and after vaccination, between prior COVID groups

		Prior COVID		P value
Time Period		Recovered COVID*	PASC*	
*Log*_*10*_ *IgG-S levels*				
Before vaccination		3.17 [2.35, 4.26]	3.25 [2.62, 3.83]	0.75
After vaccination, 0–8 weeks		4.31 [4.10, 4.52]	4.47 [4.35, 4.81]	**0.004**
More than 8 weeks after vaccination		3.74 [3.36, 4.08]	3.98 [3.59, 4.45]	**< 0.001**
*IgG-N levels*				
Before vaccination		1.42 [0.10, 3.34]	1.46 [0.49, 3.44]	0.13
After vaccination, 0–8 weeks		1.43 [0.45, 2.88]	1.86 [1.06, 3.17]	0.09
More than 8 weeks after vaccination		0.62 [0.19, 1.71]	0.82 [0.32, 1.82]	0.06
*Log*_*10*_ *(IgM-S + 1) levels*				
Before vaccination		0.29 [0.11, 0.56]	0.18 [0.08, 0.42]	0.034
After vaccination, 0–8 weeks		0.28 [0.15, 0.55]	0.18 [0.10, 0.24]	**0.001**
More than 8 weeks after vaccination		0.15 [0.09, 0.33]	0.13 [0.07, 0.28]	0.12
*ACE2 Binding levels*				
Before vaccination		100.0 [98.3, 100.0]	70.2 [14.9, 99.0]	**< 0.001**
After vaccination, 0–8 weeks		100.0 [99.0, 100.0]	100.0 [100.0, 100.0]	0.35
More than 8 weeks after vaccination		98.2 [90.7, 99.5]	99.1 [89.9, 99.9]	0.044

*Values are shown as median and interquartile range [IQR], with comparisons performed using two-sided Wilcoxon tests

**Table 3:** PASC vs. COVID-recovered status in relation to post-vaccination log10 IgG-S levels, overall and excluding individuals who received the J&J vaccine

Predictor: PASC vs. COVID-recovered status	Overall		Non-Recipients of J&J Vaccine	
	Estimate (SE)	P value	Estimate (SE)	P value
Model 0: Time from vaccination to assay*	0.20 (0.08)	0.008	0.26 (0.07)	< 0.001
Model 1: Time from vaccination to assay plus age and sex	0.15 (0.08)	0.047	0.22 (0.07)	0.002
Model 2: Adjusted for Model 1 plus Non-Hispanic White	0.16 (0.08)	0.040	0.23 (0.07)	0.002
Model 3: Adjusted for Model 1 plus Elixhauser score	0.15 (0.08)	0.046	0.22 (0.07)	0.002
Model 4: Adjusted for Model 1 plus any comorbidity	0.14 (0.08)	0.058	0.21 (0.07)	0.003

**Fig. 2:**
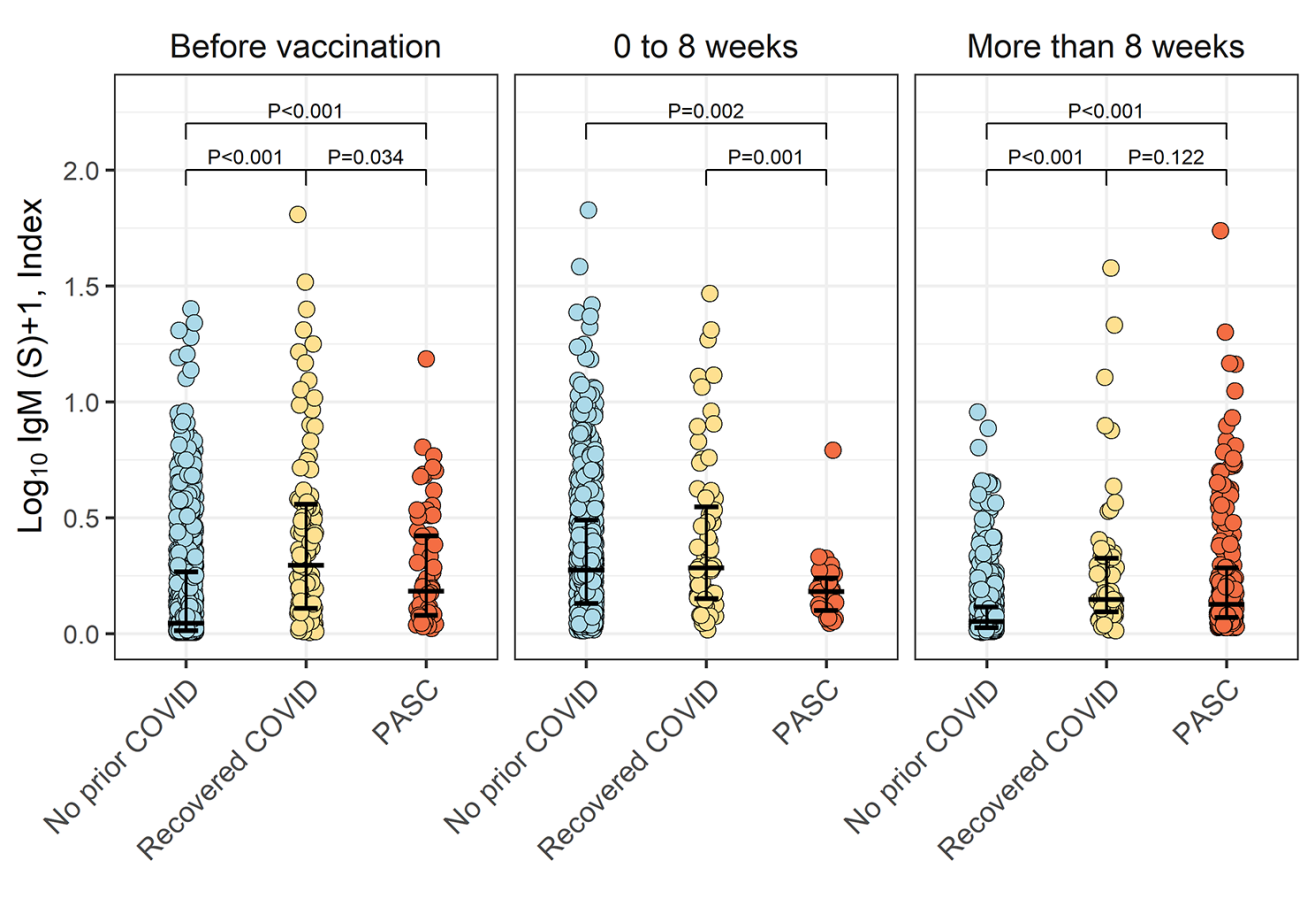
Pre- and post-vaccination IgM-S antibody levels in PASC. In both never-infected and COVID-recovered individuals, IgM-S antibody levels were expectedly increased in the immediate post-vaccination period and then significantly decreased after 8 weeks (P ≤ 0.002 for within group comparisons between the time periods before and after 8 weeks). However, in the setting of PASC, IgM-S antibody levels were not significantly lower after compared to before 8 weeks from vaccination (P = 0.33). This finding was consistent with IgM-S levels being higher in COVID-recovered than PASC-affected individuals within 8 weeks after vaccination (P = 0.001) and then not significantly different between these groups beyond 8 weeks after vaccination (P = 0.12)

Representing the more acute response to immune provocation, IgM-S antibody levels were expectedly increased in the immediate post-vaccination period and then decreased after 8 weeks (P ≤ 0.002) in the COVID-recovered individuals as well as in the referent group prior COVID (Fig. 2). However, in the setting of PASC, IgM-S antibody levels were not significantly lower even beyond 8 weeks from vaccine administration.

**Fig. 3:**
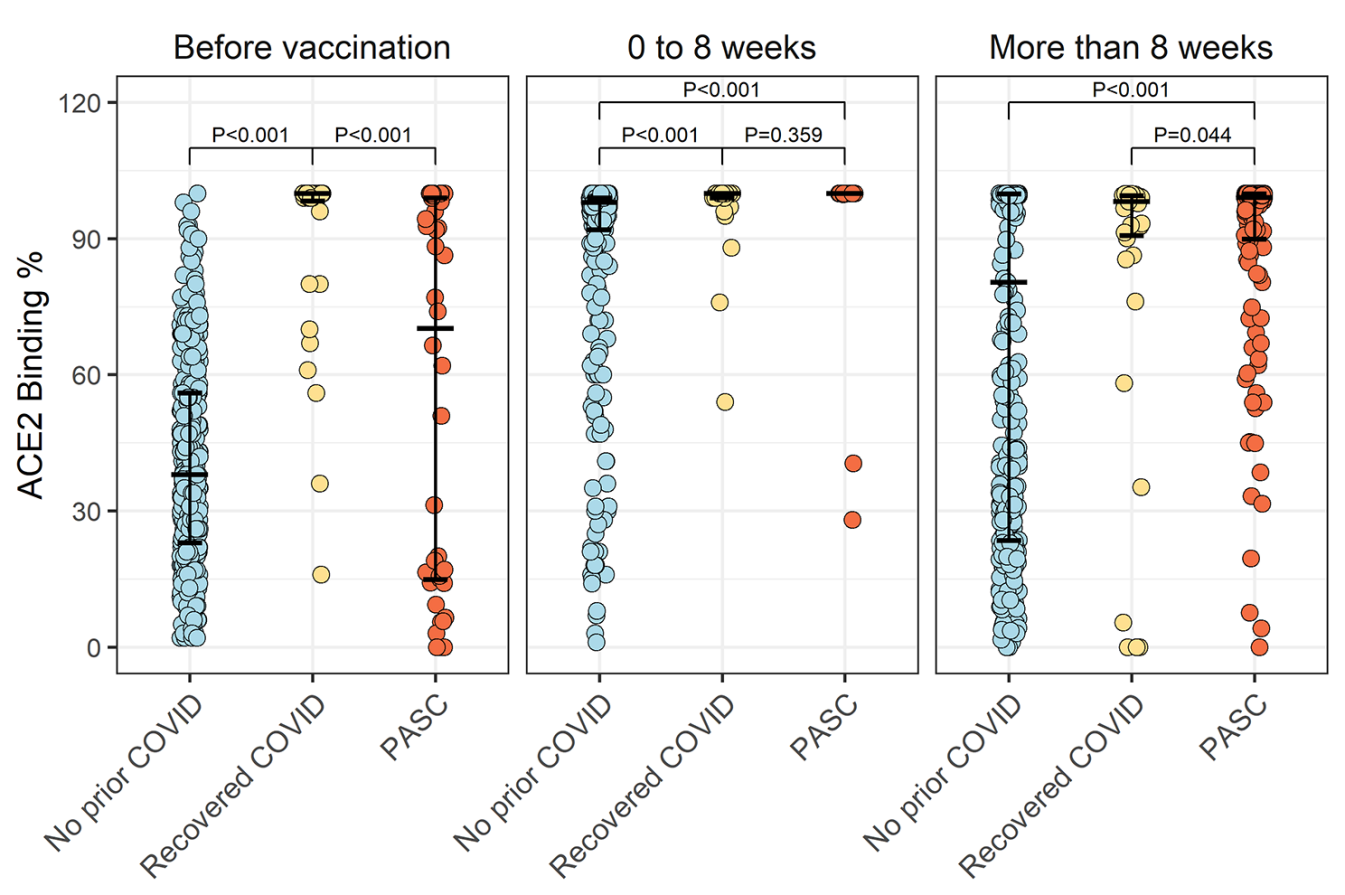
Pre- and post-vaccination ACE2 binding levels in PASC. Individuals with PASC compared to COVID-recovered individuals had similarly elevated ACE2 binding levels within 8 weeks following COVID vaccination. Notably, after 8 weeks, ACE2 binding levels remained higher in the PASC-affected compared to COVID-recovered individuals, mirroring results of IgG-S levels assessed during the same time period

We also assayed ACE2 binding inhibition levels, representing the neutralization potential of elicited receptor binding domain antibodies. In the subset of study participants with the ACE2 binding assay performed, we found results paralleled those for the IgG-S measures. Although no significant difference was seen between PASC and COVID-recovered participants during the immediate post-vaccination period, likely due to limited assays available for this timepoint, we found ACE2 binding levels were significantly higher in individuals with PASC compared to COVID-recovered (P = 0.044) more than 8 weeks after vaccination (Fig. 3; Table 2).

In secondary analyses, we found that all serological assay levels in the PASC cohort were expectedly higher than those in the referent cohort of individuals with no prior COVID (Table S4). With respect to the association of PASC versus COVID-recovered status with post-vaccine IgG-S response, we observed no significant age or sex interaction (Table S5). Notably, we did find in secondary analyses of all available serology assay data that ACE2, followed by IgM-S and IgG-S, demonstrated high AUC values for distinguishing PASC from recovered-COVID status in both crude and adjusted models (Figure S3).

## Discussion

We found evidence of persistent immune activation that differentiates individuals with PASC from COVID-recovered individuals. In particular, we observed that individuals with PASC mounted a higher IgG-S antibody response to vaccination than COVID-recovered individuals; this difference was sustained over time. Notably, this persistently elevated IgG-S response was mirrored by persistently elevated ACE2 binding levels. The significant association of ACE2 with PASC versus recovered-COVID status suggests that the aberrant immune response in PASC involves a persistent excess in not only IgG-S antibody levels but perhaps also in IgG-S neutralizing capacity. Intriguingly, we also found that while post-vaccine IgG-M antibody levels decreased over time in COVID-recovered individuals, these acute response measures remained relatively unchanged among individuals in PASC. The higher IgM-S levels seen in the COVID-recovered compared to PASC cohort may have been related to a greater proportion of participants having more recently timed infection in the former than the latter groups (Figure S2), although secondary analyses adjusting for timing of prior infection did not suggest a difference in magnitudes of effect. Together, these results indicate presence of an aberrant immune response that distinguishes PASC from recovered COVID; this aberrancy is marked by findings consistent with excess IgG-S antibody activation, in addition to a delayed or dysfunctional immunoglobulin class switching that is unmasked by vaccine provocation.

Our findings extend from studies that have characterized dynamic antibody isotype switching and persistent lymphocytic alterations in relation to severity and timing of COVID illness and recovery [13, 14]. The degree to which aberrant humoral activation may be related to an actual or perceived failure to achieve complete (systemic or organ-specific) clearance of viral antigenic material remains unclear. Accordingly, our findings also expand from recent studies revealing that COVID can lead to misdirected immune responses manifesting as excess autoantibody production, [15] with an intriguing potential predominance in males despite classic autoimmune diseases being more prevalent in females [16]. It is not yet clear if such autoreactivity tends to persist over time and mechanistically contribute to the prolonged symptomatology seen in PASC and not in COVID-recovered individuals. Nonetheless, our results indicate that some form of immune activation does indeed endure in PASC and that this phenomenon is more evident in males compared to females.

While unmasking aberrant immunity in PASC, the exact mechanisms by which vaccination provokes an augmented and more persistent IgG-S antibody response in PASC compared to COVID recovery are yet unclear. Accentuated IgG-S response could reflect persistent memory B cell activation that has been proposed as a driver of diverse autoantibody production underlying multi-systemic PASC symptomatology [17]. If true, then a relative excess in IgG-S response to an immune stressor such as vaccine could represent a common marker of PASC risk or a measure of response to therapies. Elevated IgG-S antibody response, particularly in the setting of relatively unchanged IgG-M levels, could also reflect persistent viral reservoirs across the body not identified by nasopharyngeal swabs. The finding of minimal between-group difference in IgG-N, along with overall decreasing IgG-N levels over time, argues against but does not completely rule out possible persistence of nucleocapsid antigenic exposure. Proposed immune exhaustion appears less likely, based on our data, given the robust IgG-S responses seen for most individuals with PASC. Nonetheless, the extent to which such robust response could be specific to COVID vaccines versus generalizable to other vaccine or immune challenges is unknown.

Limitations of this study include a single-center site with modest sample sizes. Further work is needed to assess generalizability of our findings in larger sized study populations. We examined humoral responses specific only to COVID vaccination, and the extent to which similar findings would be seen following other vaccine or immune challenges is unknown. We had collected post-vaccination reactogenicity data for a proportion of study participants [18] but these same data were not available for all participants; thus future work is needed to examine how post-vaccine sequelae may be related to differences in post-vaccine immune responses across PASC and non-PASC phenotypes. Given our focus on humoral immune activation, additional studies are needed to determine whether similar results would be seen for cellular responses including T cell activity. We excluded from analyses the few people in the source cohort who had received pre-exposure monoclonal antibody infusion, given its known ability to substantially increase antibody levels. Notably, there were also no participants in our cohort who received monoclonal antibody infusion for post-exposure treatment of COVID; thus, the extent to which post-vaccination antibody levels may be influenced by monoclonal antibody infusion or other treatments for an index SARS-CoV-2 infection warrants further investigation. Follow-up studies with comprehensive longitudinal data in larger cohorts are also needed to extend and validate our findings with respect to the potential sensitivity and specificity of particularly the ACE2 as well as IgM-S and IgG-S assays as tools for distinguishing PASC from non-PASC phenotypes.

## Conclusion

The accentuated and sustained serological response to vaccination in PASC has several potential near-term as well as longer-term clinical implications. Notwithstanding relatively matched elevations in ACE2 binding levels, vigorous post-vaccine IgG-S antibody response in PASC could yet signal greater, lesser, or equivocal protection against re-infection by SARS-CoV-2 and its emerging variants. Additional more comprehensive investigations are needed to assess potential variations in functional immunity, and follow-up studies will be critical for determining associated outcomes. The current analysis offers insights that complement the rich data continually emerging from other studies of PASC, while simultaneously highlighting the potential clinical utility of readily available diagnostics. More work is needed to fully evaluate the feasibility and interpretability of antibody testing in the context of clinical care for PASC patients – including possible diagnostic and prognostic applications – even as the same challenges are also evolving around the use of accessible antibody testing to guide the care of immunocompromised and other vulnerable individuals [19, 20].

## Electronic supplementary material

Below is the link to the electronic supplementary material.

Supplementary Material 1

## Data Availability

The datasets generated and/or analysed during the current study are not publicly available due to patient and participant confidentiality requirements. De-identified data requests may be available upon reasonable request from the corresponding author and will be reviewed by the Cedars-Sinai Office of Research Administration.

## Abbreviations

ACE2: Angiotensin-Converting Enzyme 2
PASC: Post-Acute Sequelae of COVID
