# Supplementary material for: Serological response to vaccination in post-acute sequelae of COVID
Source: BMC Infect Dis. 2023 Feb 16;23:97. doi: 10.1186/s12879-023-08060-y (PMC9933819; doi:10.1186/s12879-023-08060-y)
Supplement: Supplementary file 1 — Supplementary Material 1 [file 12879_2023_8060_MOESM1_ESM.docx]

**ADDITIONAL FILES**

**Additional file 1.** Supplemental tables and figures.

**Supplemental Content**

| **Table S1.** | Availability of SARS-CoV-2 serological assay data for study participants at each timepoint. |
| --- | --- |
| **Table S2.** | Symptom-related characteristics of the participants with PASC. |
| **Table S3.** | Characteristics of the study samples, including the referent group with no prior COVID. |
| **Table S4.** | Serological measures before and after vaccination across groups with and without prior COVID. |
| **Table S5.** | PASC vs COVID-recovered status in relation to post-vaccination log10 IgG-S levels, overall and by sex and age. |
|  |  |
| **Figure S1.** | Sampling strategy flow diagram. |
| **Figure S2.** | Timing of index COVID infection and study enrollment for the primary study samples. |
| **Figure S3.** | ROC analyses comparing serological assays for distinguishing PASC from COVID-recovered status. |

**Table S1.** Availability of SARS-CoV-2 serological assay data for study participants at each timepoint.

|  | **No prior COVID** | | | |
| --- | --- | --- | --- | --- |
|  | ***Total*** | Log_10_ IgG-S | Log_10_ [IgM-S+1] | ACE2 Binding |
| Before vaccination, n (%) | ***1396*** | 943 (67.6) | 943 (67.6) | 394 (28.2) |
| 0 to 8 weeks, n (%) | ***726*** | 726 (100.0) | 724 (99.7) | 304 (41.9) |
| More than 8 weeks, n (%) | ***2318*** | 2318 (100.0) | 723 (31.2) | 409 (17.6) |
|  |  |  |  |  |
|  | **Recovered COVID** | | | |
|  | ***Total*** | Log_10_ IgG-S | Log_10_ [IgM-S+1] | ACE2 Binding |
| Before vaccination, n (%) | ***170*** | 104 (61.2) | 103 (60.6) | 36 (21.2) |
| 0 to 8 weeks, n (%) | ***76*** | 76 (100.0) | 72 (94.7) | 25 (32.9) |
| More than 8 weeks, n (%) | ***231*** | 231 (100.0) | 70 (30.3) | 39 (16.9) |
|  |  |  |  |  |
|  | **PASC** | | | |
|  | ***Total*** | Log_10_ IgG-S | Log_10_ [IgM-S+1] | ACE2 Binding |
| ***All vaccine recipients*** |  |  |  |  |
| Before vaccination, n (%) | ***73*** | 73 (100.0) | 72 (98.6) | 44 (60.3) |
| 0 to 8 weeks, n (%) | ***31*** | 31 (100.0) | 31 (100.0) | 22 (71.0) |
| More than 8 weeks, n (%) | ***216*** | 216 (100.0) | 214 (99.1) | 134 (62.0) |
| ***Non-J&J vaccine recipients*** |  |  |  |  |
| Before vaccination, n (%) | ***72*** | 72 (100.0) | 71 (98.6) | 43 (59.7) |
| 0 to 8 weeks, n (%) | ***28*** | 28 (100.0) | 28 (100.0) | 19 (67.9) |
| More than 8 weeks, n (%) | ***190*** | 190 (100.0) | 188 (98.9) | 119 (62.6) |

**Table S2.** Symptom-related characteristics of the participants with PASC

| Characteristics | N=245 |
| --- | --- |
| Ongoing Symptoms |  |
| *Systemic, n (%)* |  |
| Fever | 11 (4.5) |
| Chills | 19 (7.8) |
| Fatigue | 159 (64.9) |
| Body aches | 82 (33.5) |
| Rash | 8 (3.3) |
| Dizziness/lightheadedness | 94 (38.4) |
| *Neurocognitive, n (%)* |  |
| Difficulty concentrating | 133 (54.3) |
| headache | 108 (44.1) |
| *Cardiopulmonary, n (%)* |  |
| Cough | 45 (18.4) |
| Shortness of breath | 133 (54.3) |
| Wheezing | 31 (12.7) |
| Pain with breathing | 29 (11.8) |
| Chest pain | 68 (27.8) |
| *Gastrointentinal, n (%)* |  |
| Poor appetite | 30 (12.2) |
| Nausea/vomiting | 30 (12.2) |
| Diarrhea | 27 (11.0) |
| Abdominal pain | 38 (15.5) |
| *Other, n (%)* |  |
| Sore throat | 22 (9.0) |
| Changes to smell | 64 (26.1) |
| Changes to taste | 63 (25.7) |
| Other symptoms | 136 (55.5) |
| Was working/employed prior to COVID, n (%) | 198 (80.8) |
| Returned to work since COVID, n (%) |  |
| Yes, full-time | 122 (49.8) |
| Yes, part-time | 32 (13.1) |
| No | 67 (27.3) |

**Table S3.** Characteristics of the study samples, including the referent group with no prior COVID

|  | **No Prior COVID** | **Prior COVID** | |
| --- | --- | --- | --- |
|  |  | **Recovered COVID** | **PASC** |
| N | 729 | 86 | 245 |
| Age in years, mean (SD) | 43.9 (13.1) | 42.0 (11.8) | 48.7 (13.5) |
| Age in years, range | 19 to 86 | 23 to 76 | 22 to 79 |
| Male, n (%) | 233 (32) | 23 (27) | 81 (33) |
| Non-Hispanic White, n (%) | 369 (51) | 34 (40) | 124 (51) |
| Comorbidities,* n (%) |  |  |  |
| Autoimmune disorder | 32 (4) | 2 (2) | 19 (8) |
| Cancer | 38 (5) | 3 (3) | 15 (6) |
| Chronic Pulmonary Disease | 106 (15) | 8 (9) | 1 (0) |
| Diabetes Mellitus | 26 (4) | 3 (3) | 23 (9) |
| Hypertension | 123 (17) | 10 (12) | 53 (22) |
| Elixhauser score, mean (SD) | 1.1 (2.9) | 0.7 (2.4) | 0.8 (2.9) |
| Hospitalized for COVID-19, n (%) | N/A | 0 (0) | 54 (22) |
| Post-exposure monoclonal antibody infusion, n (%) | 13 (2) | 1 (1) | 15 (6) |
| Days between infection onset and vaccination,† median [IQR] | N/A | 194 [63, 273] | 131 [106, 242] |
| SARS-CoV-2 vaccine type received, n (%) |  |  |  |
| Pfizer (monovalent) | 729 (100) | 86 (100) | 125 (51) |
| Moderna (monovalent) | 0 (0) | 0 (0) | 64 (26) |
| Johnson & Johnson | 0 (0) | 0 (0) | 22 (9) |
| Other/Unknown | 0 (0) | 0 (0) | 34 (14) |

*Comorbidities were derived from the electronic medical record using previously validated Elixhauser definitions.

†Vaccination is defined as the date of vaccine completion: second dose of a two-dose regimen, or dose of a single-dose regimen.

**Table S4.** Serological measures before and after vaccination across groups with and without prior COVID.

| Time Period | **No Prior**  **COVID*** | **Prior COVID*** | |
| --- | --- | --- | --- |
|  |  | **Recovered COVID** | **PASC** |
| *Log_10_ IgG-S levels* |  |  |  |
| Before vaccination | 0.79 [0.22, 2.91] | 3.17 [2.35, 4.26] | 3.25 [2.62, 3.83] |
| After vaccination, 0 - 8 weeks | 4.02 [3.75, 4.27] | 4.31 [4.10, 4.52] | 4.47 [4.35, 4.81] |
| More than 8 weeks after vaccination | 3.27 [2.90, 3.85] | 3.74 [3.36, 4.08] | 3.98 [3.59, 4.45] |
| *IgG-N levels* |  |  |  |
| Before vaccination | 0.02 [0.01, 0.06] | 1.42 [0.10, 3.34] | 1.46 [0.49, 3.44] |
| After vaccination, 0 - 8 weeks | 0.04 [0.02, 0.11] | 1.43 [0.45, 2.88] | 1.86 [1.06, 3.17] |
| More than 8 weeks after vaccination | 0.05 [0.03, 0.12] | 0.62 [0.19, 1.71] | 0.82 [0.32, 1.82] |
| *Log_10_ (IgM-S +1) levels* |  |  |  |
| Before vaccination | 0.05 [0.01, 0.27] | 0.29 [0.11, 0.56] | 0.18 [0.08, 0.42] |
| After vaccination, 0 - 8 weeks | 0.27 [0.13, 0.49] | 0.28 [0.15, 0.55] | 0.18 [0.10, 0.24] |
| More than 8 weeks after vaccination | 0.05 [0.03, 0.12] | 0.15 [0.09, 0.33] | 0.13 [0.07, 0.28] |
| *ACE2 Binding levels* |  |  |  |
| Before vaccination | 38.0 [23.0, 56.0] | 100.0 [98.3, 100.0] | 70.2 [14.9, 99.0] |
| After vaccination, 0 - 8 weeks | 98.0 [92.0, 99.0] | 100.0 [99.0, 100.0] | 100.0 [100.0, 100.0] |
| More than 8 weeks after vaccination | 80.4 [23.4, 99.9] | 98.2 [90.7, 99.5] | 99.1 [89.9, 99.9] |

*All values are shown as median [interquartile range].

**Table S5.** PASC vs COVID-recovered status in relation to post-vaccination log10 IgG-S levels, overall and by sex and age.

| Covariate | **Overall** | | | | **Sex** | | | | **Age** | | | |
| --- | --- | --- | --- | --- | --- | --- | --- | --- | --- | --- | --- | --- |
|  | **Crude** | | **Adjusted** | | **Male** | | **Female** | | **65 +** | | **<65** | |
|  | Est.  (SE) | P value | Est.  (SE)**^1^** | P value | Est.  (SE) **^1^** | P value | Est.  (SE)**^1^** | P value | Est.  (SE)**^1^** | P value | Est.  (SE)**^1^** | P value |
| PASC vs. COVID-recovered status | 0.20 (0.08) | 0.008 | 0.16 (0.08) | 0.040 | 0.32 (0.17)^2^ | 0.06 | 0.10 (0.08) ^2^ | 0.25 | 0.75 (0.30)^3^ | 0.027 | 0.13 (0.08)^3^ | 0.09 |
| Age | — |  | 0.01 (0.00) | 0.002 | 0.01 (0.01) | 0.12 | 0.01 (0.00) | 0.007 | — |  | — |  |
| Male sex | — |  | -0.01 (0.08) | 0.91 | — |  | — |  | 0.11 (0.24) | 0.67 | -0.05 (0.08) | 0.57 |
| Non-Hispanic White ethnicity/race | — |  | -0.08 (0.07) | 0.24 | -0.11 (0.15) | 0.45 | -0.06 (0.08) | 0.45 | -0.18 (0.27) | 0.51 | -0.06 (0.07) | 0.42 |

^1^Models adjusted for time from vaccination to IgG-S assay, age, sex, Non-Hispanic White race/ethnicity. Vaccination is defined as the date of vaccine completion (second dose of a two-dose regimen, or dose of a single-dose regimen); time from vaccination to first available antibody assay (measured more than 8 weeks after vaccination during the ‘plateau’ period) underwent spline transformation prior to entry into all multivariable regression models.

^2^For this model, the multiplicative sex interaction term was non-significant at P=0.17.

^3^For this model, the multiplicative age interaction term was non-significant at P=0.06.

**Figure S1.** Sampling strategy flow diagram.

**Figure S2.** Timing of index COVID infection and study enrollment for the primary study samples. Colored bars represent time periods when one or more particular types of variants were predominant according to regional public health surveillance data (e.g. Mixed, Alpha, Delta [D], Omicron [Omicr]).

**
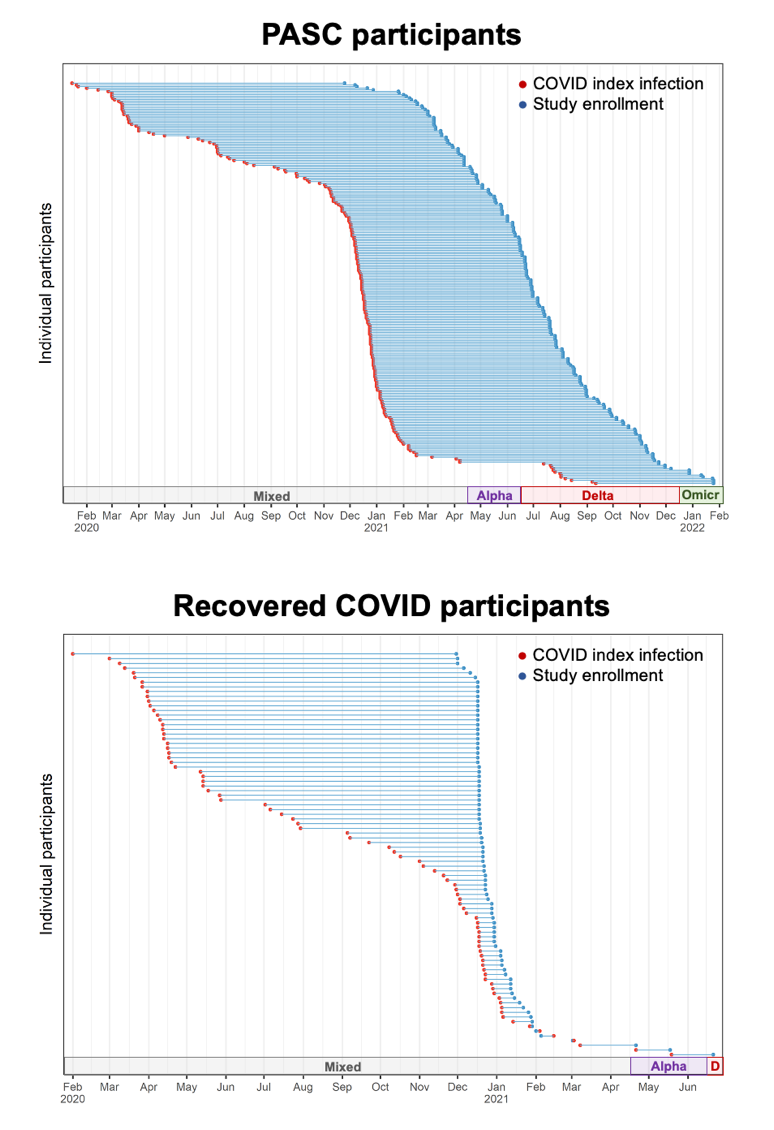
**

**Figure S3.** ROC analyses comparing serological assays for distinguishing PASC from COVD-recovered status. In ROC analyses of available data collected from all PASC and COVID-recovered participants at all timepoints, the AUC was highest for ACE2 followed by IgM-S and then IgG-S in both crude and adjusted models. Crude models accounted for time between vaccination and the assay; adjusted models included age, sex, race/ethnicity, and Elixhauser comorbidity index as covariates.
